# Oral Health-Related Quality of Life in Young Adults: A Survey of Russian Undergraduate Students

**DOI:** 10.3390/ijerph15040719

**Published:** 2018-04-11

**Authors:** Sergei N. Drachev, Tormod Brenn, Tordis A. Trovik

**Affiliations:** 1 Department of Community Medicine, Faculty of Health Sciences, UiT The Arctic University of Norway, N-9037 Tromsø, Norway; tormod.brenn@uit.no (T.B.); tordis.a.trovik@uit.no (T.A.T.); 2 International School of Public Health, Northern State Medical University, 163000 Arkhangelsk, Russia

**Keywords:** oral health-related quality of life, medical and dental students, North-West Russia

## Abstract

Background: Oral health (OH) is poor among young adults in Russia, but there is little information on OH-related quality of life (OHRQoL) in this population. We investigated how socio-demographic factors, self-reported OH characteristics, oral health behaviour, and clinically-assessed OH are related to OHRQoL in medical and dental students in North-West Russia. Methods: This cross-sectional study included 391 medical and 275 dental Russian undergraduate students aged 18–25 years. Information on socio-demographic, self-reported OH characteristics, and oral health behaviour was obtained from a structured, self-administered questionnaire. A clinical examination was performed to assess dental caries experience based on the decayed (D) missing (M) filled (F) teeth (T) index; Simplified Oral Hygiene Index; and Gingival Index. OHRQoL was measured by the OH Impact Profile (OHIP-14). Results: 53.6% of students reported low OHRQoL during the last 12 months. Female sex (odds ratio [OR] = 1.48, 95% confidence interval [CI]: 1.00–2.19), rural place of childhood residence (OR = 1.56, 95% CI: 1.06–2.28), poor self-assessed dental aesthetic (OR = 1.75, 95% CI: 1.16–2.64), dissatisfaction with mouth and teeth (OR = 2.51, 95% CI: 1.68–3.77), and DMFT index (OR = 1.05, 95% CI: 1.01–1.09), were all significantly, independently associated with low OHRQoL. Conclusion: Socio-demographic factors (rural place of childhood residence, female sex), poor self-reported OH characteristics, and high DMFT index were associated with low OHRQoL.

## 1. Introduction

Dental caries and periodontal diseases are the most common oral diseases, affecting millions of people worldwide. In addition to objective methods of oral health (OH) evaluation performed by dental professionals, patient perception of oral disease is also important in the assessment of treatment needs and clinical outcome [1,2]. The concept of OH-related quality of life (OHRQoL) uses patient-centred outcome measures to identify the impact of OH on aspects of everyday life in terms of a person’s functional, social, and psychological well-being [3]. Over the past decades, a set of psychometric instruments has been developed to assess OHRQoL. The OH Impact Profile (OHIP) is widely used to measure OHRQoL in adults and dentate elderly people [2]. The short version of the OHIP includes 14 items (OHIP-14), which are based on Locker’s conceptual model for measuring OH [2,4,5]. These items represent the consequences of oral diseases and the negative impact they have on OHRQoL. The validity and reliability of OHIP-14 has been shown in many studies, and the instrument has been translated into several languages, including Russian [3,6]. 

Studies have shown that young and middle-aged adults report worse OH than older adults, despite the fact that oral problems tend to increase with age [7,8,9]. The factors that affect self-reported OH are not well understood, but it has been suggested that oral diseases have a deleterious effect on subjective OH, and that this effect is likely higher at younger ages [9]. Moreover, the attitude toward OH acquired in young life manifests as life goes on and may affect OHRQoL. Several studies on OHRQoL in young adult populations, including young university students, have been conducted in Japan [10], Sweden [11], Tanzania [12], Malaysia [13], Australia [14], China [15], and Korea [16]. Previously reported factors associated with OHRQoL include negative life events [14]; education [12,13]; self-rated OH [10,12]; and subjective symptoms of temporomandibular disorders and oral pain [10]. The influence of clinical factors (dental caries, missing teeth, and periodontal status) on OHRQoL is inconsistent, with some studies showing no relationship [11,15] and others showing that poor clinically-assessed OH is associated with worse OHRQoL [10,12,16]. It was also found that malocclusion has a negative impact on OHRQoL in young adults [13,16]. Few studies on OHRQoL targeted dental students [17,18,19]. Medical and dental students are expected to be more conscious of health-related issues, including dental health. Moreover, these students tend to have a higher socioeconomic background, which in turn, may lead to better self-reported OH and clinically-assessed OH and, eventually, to a higher OHRQoL. Nevertheless, in a Brazilian study nearly half of participating dental students reported negative impacts on their OHRQoL [17]. Self-reported OH problems and aspects related to previous dental experience were found to have a greater impact on OHRQoL [17,18], although no clinical factors were studied. Almost all of the aforementioned studies used OHIP-14 to measure OHRQoL in young adult populations. 

To our knowledge, there has been little research on OHRQoL in Russian populations. Nonetheless, in 2007, Barer et al. validated the Russian version of OHIP-14 in patients with evidence of chronic generalized periodontitis [6]. The English version of OHIP-14 was translated/back-translated into Russian/English by two bilingual persons, independently. The final version of the questionnaire was developed, pilot-tested on 25 middle-aged Russian adults (8 men, 17 women), and published in Russian [6]. The psychometric properties of the instrument were examined, and the authors reported good face and content validity of the OHIP-14 items. Another Russian study assessed the effectiveness of periodontal treatment on OHRQoL in patients with various forms of periodontitis [20]. We found no epidemiological studies that assessed OHRQoL in Russian young adults. Nevertheless, OH has been observed to be poor in this age group in Russia; high prevalence of dental caries and high dental caries experience were found among students aged 16–25 years in Moscow [21] and among medical and dental students aged 18–25 years in Arkhangelsk [22].

The aim of this study was to investigate how socio-demographic factors, self-reported OH characteristics, oral health behaviour, and clinically-assessed OH are related to OHRQoL measured by OHIP-14 in medical and dental students in North-West Russia.

## 2. Materials and Methods

### 2.1. Study Setting and Population

This cross-sectional study was conducted at the Northern State Medical University (NSMU), Arkhangelsk, North-West Russia, during the 2015–2016 academic year. Approximately 3900 students, mainly from the European North-West of Russia, which includes the regions of Arkhangelsk, Murmansk and Vologda; the Komi and Karelia Republics; and the Nenets Autonomous Okrug attended the NSMU. We invited full-time undergraduate students from two faculties: (1) medical (*n* = 1482), which included students from the departments of general medicine and paediatric medicine; and (2) dental (*n* = 524). For convenience, students from other non-medical and smaller medical faculties and departments of the NSMU (medical biochemistry, pharmacy, and medical prophylaxis) were not invited, nor were students from the international faculty of general practitioners, as the study targeted Russian students only.

### 2.2. Sampling

The study included two stages. In Stage 1, all students from the medical and dental faculties and each year of education (6 and 5 years for medical and dental students, respectively) were informed about the study and invited to participate at the end of a scheduled classroom lecture. Altogether, 1579 students (78.7%) attended the recruitment lectures, of whom 1385 (87.7%) agreed to participate, signed the informed consent, completed a self-administered questionnaire in Russian, and gave their mobile phone number so they could be contacted for Stage 2. 

All dental students (*n* = 420) participating in Stage 1 and a stratified, random, proportionate sample of medical students (*n* = 823) were invited to Stage 2. Altogether, 62 students refused to participate in Stage 2, 135 students did not answer their phone at two separate calls on two separate days, and 145 students did not attend Stage 2. We also excluded 94 students who were outside the target age (18–25 years), were not of Russian nationality, had fixed orthodontic bands, or were pregnant. A total of 807 students (overall response rate of 64.9%) agreed to participate in Stage 2, completed a second, self-administered questionnaire in Russian, and underwent a clinical dental examination. The students with no missing data (*n* = 666) were included in statistical analysis. Details regarding the sample size calculation for Stage 2 have been described previously [22]. 

### 2.3. Questionnaires

The Stage 1 questionnaire collected information on socio-demographic variables, which included age group (18–20/21–25 years), sex, faculty (medical/dental), and place of childhood residence (urban/rural). The questionnaire also collected information on self-assessed OH, self-assessed dental aesthetic, satisfaction with mouth and teeth, and oral health behaviour. Self-assessed OH and self-assessed dental aesthetic were dichotomized as “good” (excellent, very good, good) and “poor” (fair, poor). The applied cut-off level reflects public health perspectives and treatment needs, rather than detailed individual statements of symptoms. Satisfaction with mouth and teeth was assessed by one item with response options “yes”, “no”, and “difficult to answer”. Questions on oral health behaviour included regularity of dental visits (irregular, i.e., occasionally/no visits during the last 3 years; or regular, i.e., at least once every 6 months/at least once a year) and using toothpaste with fluoride (without fluoride/difficult to answer; or with fluoride). Frequency of tooth-brushing was categorized as infrequent (never/less than once a week/once every few days/once a day) and frequent (twice a day/more than twice a day). Moreover, students were asked to report how often they skipped tooth-brushing. Responses were given on a 3-point scale: (1) never or almost never, (2) sometimes during a week, and (3) every day or almost every day. For analysis, the variable “skipping tooth-brushing” was dichotomized as no (1) and yes (2,3). 

The Stage 2 questionnaire gathered information on subjective socioeconomic status (SES), mother’s education, and included the OHIP-14 to measure OHRQoL. The respondents rated the SES of their family (according to education, income, and occupation) using the 10-step MacArthur Scale of Subjective Social Status, for which 10 was “best off” and 1 was “worst off” [23]. The median SES (6.0) was used as the cut-off, and SES was dichotomized as “low” (from 1 to 5) and “high” (from 6 to 10). Mother’s education was categorized as lower than university (high school: 9–11 years of school; specialized secondary: professional medical or pedagogical college, technicum), and university.

The questions on self-assessed OH, dental aesthetic, and regularity of dental visits in the Stage 1 questionnaire and the question on mother’s education in the Stage 2 questionnaire had the response option “difficult to answer”. When that response was chosen (*n* = 31 and *n* = 7 in the Stage 1 and 2 questionnaires, respectively), this data was considered missing and the students were excluded from the analysis.

#### Oral Health-Related Quality of Life as Measured by the Oral Health Impact Profile-14: Validity and Reliability

OHRQoL was measured by the Russian version of the OHIP-14, which was previously validated and published in Russian [6]. The same items were used in the present study without any modifications (Table S1 in the Supplementary Materials). The instrument considers seven dimensions of negative impact on OHRQoL: functional limitation, physical pain, psychological discomfort, physical disability, psychological disability, social disability, and handicap. There are two items for each dimension, for a total of 14 items. Participants rated the frequency with which they experienced each of these items in the last 12 months using a 5-point Likert scale (“never” = 0, “hardly ever” = 1, “occasionally” = 2, “fairly often” = 3, and “very often” = 4). In addition, each item had the response option “I do not know”. When that response was chosen for at least one item, the data was considered missing, and the student was excluded from the analysis. 

The severity of impact on OHRQoL was determined by computing the sum of all items in the OHIP-14, with a maximum possible score of 56 points. A higher score indicated a lower OHRQoL. Based on clinical relevance, the prevalence of low OHRQoL was defined as the proportion of students who responded “occasionally”, “fairly often”, or “very often” for at least one item on the OHIP-14, as was previously applied in other studies among young populations [14,15,17]. 

In the present sample, OHIP-14 scores discriminated significantly between students with good self-assessed OH (mean OHIP-14 score 3.6) and poor self-assessed OH (mean OHIP-14 score 6.6), thus demonstrating good construct validity. Cronbach’s alpha based on standardized items was 0.85, indicating good internal consistency of the OHIP-14. The average inter-item correlation for the OHIP-14 items was 0.28 (range: 0.10–0.66), with no negative correlations. The corrected item–total correlations ranged from 0.27 to 0.66, and all values were above the minimum recommended level of 0.20 for including an item into a scale [24].

### 2.4. Clinical Dental Examination

From February to May 2016, a clinical dental examination was performed by one dentist (SND), calibrated to World Health Organization standards [25]. Dental caries was detected visually, and no radiographs were taken. All permanent teeth, excluding third molars, were taken into consideration to measure dental caries experience by the DMFT index, which is the sum of decayed teeth (DT), missing teeth due to caries (MT), and filled teeth (FT). The Simplified Oral Hygiene Index (OHI-S) was calculated as the sum scores of the average individual amount of debris (range 0–3) and calculus (range 0–3) found on the preselected tooth surfaces on four posterior and two anterior teeth [26]. To assess the qualitative changes in gingival soft tissue, the Gingival Index (GI) was applied [27]: four areas (mesial, distal, buccal, and lingual) of each of the six index teeth (44/32/36/24/12/16) were examined to calculate GI. In order to test reliability, 54 randomly-selected students underwent another clinical examination in June 2016. Intraclass correlation coefficients for DMFT and GI were 0.989 (95% confidence interval (CI): 0.981–0.993) and 0.828 (95% CI: 0.721–0.896), respectively. 

### 2.5. Statistical Analysis

The chi-square test was applied to compare the proportion of students with/without low OHRQoL between categories of socio-demographic factors, self-reported OH characteristics, and oral health behaviour. When comparing data on clinically-assessed OH (DMFT index, OHI-S index, GI), the Mann–Whitney U test was used for the two independent groups (with and without low OHRQoL). 

Multivariable binary logistic regression with robust estimates was used, with the dichotomized dependent variable coded as 0 = without low OHRQoL and 1 = with low OHRQoL, and independent variables that showed *p*-values < 0.2 in univariable analysis. Backward stepwise selection was used to find significant associations, and levels for removal and addition to the final model were applied as 0.2 and 0.1, respectively. Data was analysed with IBM SPSS Statistics for Macintosh version 23.0 (IBM Corp., Armonk, NY, USA) and STATA version 14.0 (StataCorp, College Station, TX, USA). *p*-values < 0.05 were considered as statistically significant.

### 2.6. Ethical Considerations

All students participating in the study gave their verbal and written informed consent at Stage 1. The participants were informed that they could withdraw from the study at any time. Ethical approval for this study was obtained from the Regional Ethical Committee of Norway (2015/1788/REK nord) and the Ethical Committee of the NSMU, Russia (№ 05/10-15 from 19.10.2015). 

## 3. Results

Of the 807 students who answered the OHIP-14, 20 omitted one item, one did not answer all items, and 57 students chose the response option “I do not know” for at least one item. There were no significant differences across socio-demographic variables between students without missing OHIP-14 data (*n* = 729) and those with missing data (*n* = 78). Nevertheless, students with missing data more often reported poor self-assessed dental aesthetic, dissatisfaction with mouth and teeth/or difficult to answer, and had poor clinically-assessed OH (high DMFT, MT, and OHI-S). 

**Figure 1:**
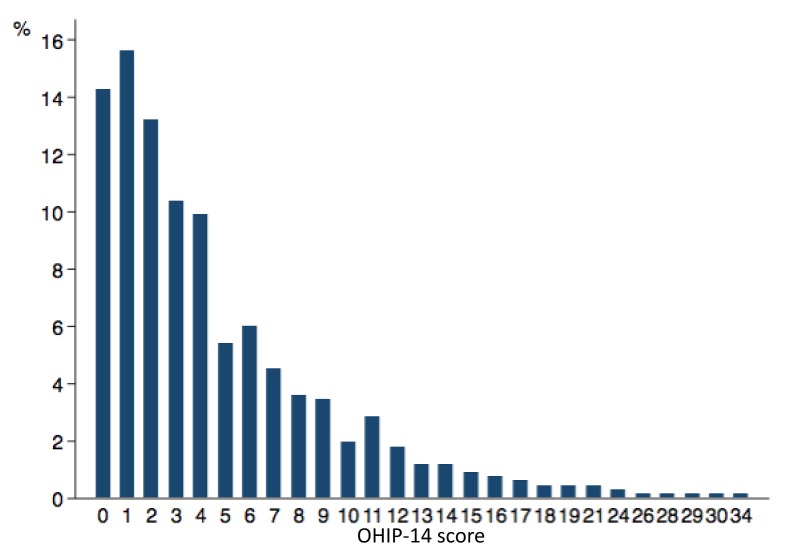
Histogram of the Oral Health Impact Profile 14 (OHIP-14) score in the overall study sample (*n* = 666).

**Figure 2:**
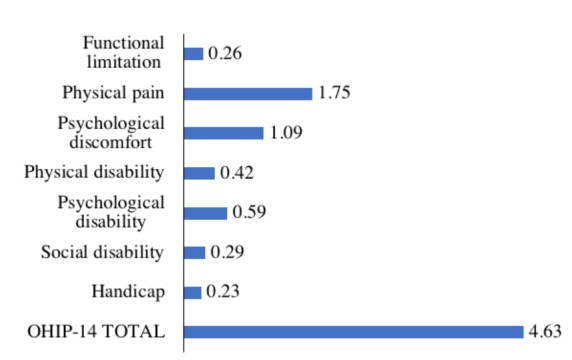
Mean Oral Health Impact Profile 14 (OHIP-14) score (by dimensions and total) in the study sample (*n* = 666).

A total of 666 students were included in the statistical analysis, and the mean OHIP-14 score was 4.63 (standard deviation [SD] 4.90; range = 0–34) (Figure 1). More than half of the students (53.6%) reported low OHRQoL; the mean number of items with a reported frequency of “occasionally” or more often was 1.27 (SD = 1.77; range = 0–11). The highest mean scores were observed for the dimensions physical pain and psychological discomfort (Figure 2), which were also the most frequently reported dimensions with impact on OHRQoL (Table 1). With respect to single OHIP-14 items, the prevalence of low OHRQoL varied from 1.7% (for the item “unable to function” in the dimension handicap) to 37.0% (for the item “painful aching in mouth” in the dimension physical pain).

Mean age of the students was 20.2 years (SD 1.6); 75.4% were women, 71.9% reported urban place of childhood residence, and 53.8% had mother with a university education. The prevalence of low OHRQoL was higher in older students than in younger students; in females than in males; in medical students than in dental students; and in those who reported rural place of childhood residence than in those who reported urban place of childhood residence. No differences in the proportion of students with low OHRQoL were observed between categories of subjective SES or mother’s education. Nearly two-thirds of the students had good self-assessed OH and self-assessed dental aesthetic, while there was an approximately equal number of students who were satisfied and dissatisfied with their mouth and teeth. Students with poor self-assessed OH, poor self-assessed dental aesthetic, and who reported dissatisfaction with mouth and teeth were more frequently in the group with low OHRQoL (Table 2).

When looking at oral health behaviour, 77.0% and 47.0% of the students reported regular dental visits and using a toothpaste with fluoride, respectively. Although 80.2% of the students reported frequent tooth-brushing, 33.3% reported skipping tooth-brushing sometimes during a week, every day, or almost every day. No differences in the proportion of students with low OHRQoL were observed between categories of regularity of dental visits, using toothpaste with fluoride, and tooth-brushing. Students who reported skipping tooth-brushing were more frequently in the group with low OHRQoL (Table 3). 

The mean DMFT index was 7.46 (SD 4.43), with FT accounting for 90.6% of the dental caries experience. The mean OHI-S index and GI was 1.09 (SD 0.50) and 0.27 (SD 0.24), respectively. A higher number of DT, MT, FT, high DMFT index, and high OHI-S index were associated with low OHRQoL (Table 4). 

Multivariable logistic regression with the dependent binary variable showed that female sex, rural place of childhood residence, poor self-assessed dental aesthetic, dissatisfaction with mouth and teeth, and high DMFT index were associated with higher odds of having low OHRQoL. For instance, the odds of having low OHRQoL among students with poor self-assessed dental aesthetic was 1.75 (95% CI: 1.16–2.64) times higher than that found in those with good self-assessed dental aesthetic after adjustment for other variables in the model. The most important predictors of low OHRQoL were satisfaction with mouth and teeth and self-assessed dental aesthetic. All independent variables in the final model explained 20.6% of the variation in the dependent variable (Table 5).

## 4. Discussion

### 4.1. Main Findings

The present study found that more than half of the medical and dental students aged 18–25 years attending the NSMU in Arkhangelsk, North-West Russia had low OHRQoL. Socio-demographic factors (rural place of childhood residence, female sex), poor self-reported OH characteristics, and high DMFT index, were associated independently with low OHRQoL.

### 4.2. Data Interpretation and Comparisons with Previous Studies

The severity (mean OHIP-14 score) and prevalence of low OHRQoL in medical and dental students in the present study (4.6 and 53.6%) are similar to that reported in Brazilian dental students (4.5 and 45.0%) [17] and Chinese young adults (6.3 and 50.6%) [15]. By contrast, an Indian study found a mean OHIP-14 score of 13.4 and 10.7 in dental students in their first and fourth year of education, respectively [18], while a Japanese study reported a mean OHIP-14 score of 1.9 in first-year university students [10]. Direct comparison of these results with our data must be done with caution. Evaluation of quality of life, including OHRQoL, depends on an individual’s expectations and experiences, which vary according to social, psychological, socioeconomic, demographic, and other cultural factors [28]. Someone with poor OH and low expectations may not consider themselves to have low OHRQoL and report being satisfied. By contrast, individuals who have good OH and high expectations may experience low OHRQoL, due to even minor oral problems and report being dissatisfied [28]. Previous studies showed that 80.0% of Brazilian dental students were satisfied with their mouth and teeth [17]; only 15.1% of Chinese young adults [15] and 36.8% of Japanese university students [10] reported good OH, while 44.4% and 63.8% of our medical and dental students were satisfied with their mouth and teeth and reported good OH, respectively. To compare these results, we need to know the frames of reference, i.e., the expectations and experiences these people used, when assessing their OH, satisfaction, and OHRQoL. Qualitative research should be designed to answer these questions [29]. Nevertheless, in the present study, we found that the OHIP-14 dimensions of physical pain and psychological discomfort were the biggest drivers of low OHRQoL, which is in line with all aforementioned studies [10,15,17,18]. Therefore, one may assume a similar pattern of OHRQoL exists in young adults in different countries. 

We found that the strongest factors associated with low OHRQoL were poor self-reported OH characteristics. This was expected and is in line with results from other studies [10,15,17,18]. One obvious explanation is that the concept of OHRQoL is based on outcome measures from the patients’ perspective, rather than from a dental professional’s viewpoint [1,2,3]. Indeed, dissatisfaction with mouth and teeth and poor self-assessed dental aesthetic may best reflect the OHIP-14 dimensions of psychical pain and psychological discomfort, which were the biggest drivers of low OHRQoL in our study. Physical pain is often considered easy to remember [17]. Psychological discomfort may result from poor dental aesthetic and dissatisfaction with mouth and teeth; a Malaysian study showed that psychological discomfort had the highest reported impact on OHRQoL in young adults with malocclusion [13]. These findings may have important implications in dental practice by allowing dentists to assume the OHRQoL of young adults asking them about their dental aesthetic and satisfaction with their mouth and teeth. 

In our study, a higher DMFT index was associated with low OHRQoL. In contrast, a Swedish study did not find any differences in OHRQoL between young adults at high risk (DMFT > 8) and low risk (DMFT = 0) of caries [11]; nor were differences in DMFT index found in young adults in China [15]. Nevertheless, Japanese university students with a higher DMFT index had lower OHRQoL [10]. In the present study, the mean DMFT index was 7.5, while in China and in Japan, the corresponding values were 1.4 [15] and 2.0 [10], respectively. At present, the mechanisms of the relationship between dental caries experience and OHRQoL are unclear [10]. Given that physical pain was the OHIP-14 dimension most frequently reported, one may assume that the dental caries experience in our medical and dental students was likely associated with pain in mouth. Public health measures, as well as dental practitioners, should focus on the prevention of dental diseases to decrease dental pain and DMFT index and improve OHRQoL in young Russian adults. 

Our study also showed that students who lived in rural places during childhood had higher odds of reporting low OHRQoL compared to those who lived in urban places. Geographical remoteness, socioeconomic deprivation, and limited access to OH services have been discussed by other researchers to explain these differences [30]. Indeed, the European North-West of Russia has a low population density, covering an area of approximately 1.5 million km^2^, but with a population of only 4.6 million (78.9% urban in 2016) [31]. In addition, the inhabitant-to-dentist ratio in North-West Russia is high; much higher, for example, than in the neighbouring Nordic countries (2294 inhabitants per dentist in North-West Russia vs. 1262 in Norway and 1101 in Sweden) [32]. The corresponding figure in rural areas of North-West Russia is even higher (~3700 inhabitants per dentist in the Arkhangelsk Region) [33].

Female students showed higher odds of having low OHRQoL than male students. One possible explanation is that women are more likely to report more severe and frequent pain than men, although mechanisms behind this phenomenon remain understudied [34]. Moreover, one may speculate that women are more concerned about their appearance, and thus may describe their psychological discomfort more openly than men. Nevertheless, other studies found no sex differences in OHRQoL in young adults [13,14,15,16,17,19]. 

### 4.3. Strengths of the Study

This is the first study in North-West Russia to investigate OHRQoL and its associated factors in young adults aged 18–25 years. We applied the Russian version of the OHIP-14, an instrument commonly used for adults and elderly people, which was validated in another adult Russian population [6]. Although the instrument was validated among middle-aged adults with periodontal diseases, the results of the present study also provide evidence of good internal consistency, with sufficient face and construct validity of the OHIP items when applied to young adults. Along with self-assessed OH outcomes, clinical dental examinations were performed on all study participants, and reliability tests showed good consistency of the obtained clinical data.

### 4.4. Limitations of the Study

Due to the cross-sectional study design, no causal relationships in the association between OHRQoL and the factors studied or trends in the prevalence of low OHRQoL over time can be determined. Only medical and dental students from the NSMU participated in the study, which may limit the generalization of our findings to the young Russian population at large in North-West Russia. One may speculate that medical and dental students are a fortunate group of young adults in terms of SES and general and oral health-related issues. Nevertheless, in the present study, the subjective SES values students reported were close to average (median was 6.0 on the MacArthur Scale). In addition, one-third of the students reported skipping tooth-brushing, which, to some extent, may reflect poor oral health behaviour. The OHIP-14 scores may be positively overestimated due to the 64.9% response rate for Stage 2. Moreover, students who were excluded due to missing data in the OHIP-14 (9.7%) more often had poor self-assessed dental aesthetic, dissatisfaction with their mouth and teeth, and poor clinically-assessed OH, which might have biased our ORs, resulting in an underestimation of the OR estimates. Only visual and tactile methods were applied during the clinical dental examination; radiographs were not taken, which could lead to an underestimation of dental caries. Information on SES and dental aesthetic was self-reported; thus, the possibility of social desirability bias due to under- or over-reporting cannot be ruled out. 

## 5. Conclusions

OH affects the quality of life of medical and dental students aged 18–25 years attending the NSMU in Arkhangelsk, North-West Russia. Physical pain and psychological discomfort were the most frequently reported OHIP-14 dimensions with impact on OHRQoL. Poor self-reported OH characteristics (poor self-assessed dental aesthetic and dissatisfaction with mouth and teeth) were the strongest factors associated with low OHRQoL. Clinically-assessed OH (high DMFT index) and socio-demographic factors (female sex, rural place of childhood residence) were also found to be significant predictors of low OHRQoL in medical and dental students of the NSMU. Public health measures should focus on the prevention of dental caries and the development of strategies to promote oral health in young Russian adults, specifically in those who live in rural areas. 

## Figures and Tables

**Table 1:** Frequency of responses to items in the Oral Health Impact Profile 14 (OHIP-14) in the study sample.

Dimension	Item	*n* (%)				
		Never (OHIP-14 = 0)	Hardly Ever (OHIP-14 = 1)	Occasionally (OHIP-14 = 2)	Fairly Often (OHIP-14 = 3)	Very Often (OHIP-14 = 4)
Functional limitations	Trouble pronouncing words	562 (84.4)	77 (11.6)	21 (3.2)	5 (0.8)	1 (0.2)
	Worsened sense of taste	637 (95.6)	23 (3.5)	4 (0.6)	2 (0.3)	0 (0.0)
Physical pain	Painful aching in mouth	164 (24.6)	256 (38.4)	225 (33.8)	17 (2.6)	4 (0.6)
	Uncomfortable to eat food	410 (61.6)	139 (20.9)	98 (14.7)	17 (2.6)	2 (0.3)
Psychological discomfort	Being self-conscious	465 (69.8)	95 (14.3)	83 (12.5)	16 (2.4)	7 (1.1)
	Feeling tense	414 (62.2)	144 (21.6)	84 (12.6)	17 (2.6)	7 (1.1)
Physical disability	Unsatisfactory diet	597 (89.6)	54 (8.1)	11 (1.7)	3 (0.5)	1 (0.2)
	Interrupting meals	522 (78.4)	103 (15.5)	36 (5.4)	4 (0.6)	1 (0.2)
Psychological disability	Difficulty relaxing	559 (83.9)	77 (11.6)	25 (3.8)	3 (0.5)	2 (0.3)
	Embarrassed	509 (76.4)	85 (12.8)	58 (8.7)	10 (1.5)	4 (0.6)
Social disability	Irritable with other people	593 (89.0)	51 (7.7)	20 (3.0)	1 (0.2)	1 (0.2)
	Difficulty doing usual jobs	593 (89.0)	51 (7.7)	20 (3.0)	2 (0.3)	0 (0.0)
Handicap	Life less satisfying	594 (89.2)	49 (7.4)	22(3.3)	1 (0.2)	0 (0.0)
	Unable to function	624 (93.4)	31 (4.7)	10 (1.5)	1 (0.2)	0 (0.0)

**Table 2:** Socio-demographic and self-reported oral health characteristics of the study sample and proportion of students with low oral health-related quality of life (OHRQoL).

Variable	*n* (%)	Low OHRQoL ^1^, *n* (%)	*p* ^2^
**Age group (years)**			0.027
18–20	390 (58.6)	195 (50.0)	
21–25	276 (41.4)	162 (58.7)	
**Sex**			0.004
Male	164 (24.6)	72 (43.9)	
Female	502 (75.4)	285 (56.8)	
**Faculty**			<0.001
Medical	391 (58.7)	232 (59.3)	
Dental	275 (41.3)	125 (45.5)	
**Place of childhood residence**			0.011
Urban	479 (71.9)	242 (50.5)	
Rural	187 (28.1)	115 (61.5)	
**Subjective SES**			0.323
Low (less than 6.0)	222 (33.3)	125 (56.3)	
High (6.0 and more)	444 (66.7)	232 (52.3)	
**Mother’s education**			0.445
<University	308 (46.2)	170 (55.2)	
University	358 (53.8)	187 (52.2)	
**Self-assessed oral health**			<0.001
Good	425 (63.8)	187 (44.0)	
Poor	241 (36.2)	170 (70.5)	
**Self-assessed dental aesthetic**			<0.001
Good	415 (62.3)	180 (43.4)	
Poor	251 (37.7)	177 (70.5)	
**Satisfaction with mouth and teeth**			<0.001
Yes	296 (44.4)	109 (36.8)	
No	279 (41.9)	196 (70.3)	
Difficult to answer	91 (13.7)	52 (57.1)	

Abbreviations: OHIP-14 Oral Health Impact Profile 14; SES Socioeconomic status. ^1^ Low OHRQoL is defined as the proportion of students who responded “occasionally”, “fairly often”, or “very often” for at least one item on the OHIP-14; ^2^ *p* from the chi-square test.

**Table 3:** Oral health behaviour of the study sample and proportion of students with low oral health-related quality of life (OHRQoL).

Variable	*n* (%)	Low OHRQoL ^1^, *n* (%)	*p* ^2^
**Regularity of dental visits**			0.462
Irregular	153 (23.0)	86 (56.2)	
Regular	513 (77.0)	271 (52.8)	
**Toothpaste**			0.457
Without fluoride/difficult to answer	353 (53.0)	194 (55.0)	
With fluoride	313 (47.0)	163 (52.1)	
**Tooth-brushing**			0.527
Infrequent	132 (19.8)	74 (56.1)	
Frequent	534 (80.2)	283 (53.0)	
**Skipping tooth-brushing**			0.021
Yes	222 (33.3)	133 (59.9)	
No	444 (66.7)	224 (50.5)	

Abbreviations: OHIP-14 Oral Health Impact Profile 14. ^1^ Low OHRQoL is defined as the proportion of students who responded “occasionally”, “fairly often”, or “very often” for at least one item on the OHIP-14; ^2^ *p* from the chi-square test.

**Table 4:** Clinically-assessed oral health variables among students with and without low oral health-related quality of life (OHRQoL).

Variable	Without Low OHRQoL ^1^	With Low OHRQoL ^1^	*p* ^2^
	Mean (SD)	Mean (SD)	
**DT**	0.49 (1.02)	0.69 (1.25)	0.020
**MT**	0.07 (0.29)	0.15 (0.47)	0.017
**FT**	6.08 (3.98)	7.34 (4.18)	<0.001
**DMFT**	6.63 (4.14)	8.18 (4.55)	<0.001
**OHI-S**	1.04 (0.51)	1.14 (0.49)	0.012
**GI**	0.26 (0.24)	0.28 (0.24)	0.082

Abbreviations: OHIP-14 Oral Health Impact Profile 14; DT decayed teeth; MT missing teeth due to caries; FT filled teeth; DMFT decayed, missing and filled permanent teeth; OHI-S Simplified Oral Hygiene Index; GI Gingival Index; SD standard deviation; ^1^ Low OHRQoL is defined as the proportion of students who responded “occasionally”, “fairly often”, or “very often” for at least one item on the OHIP-14; ^2^ *p* from the Mann-Whitney U test.

**Table 5:** Adjusted odds ratio of having low oral health-related quality of life in the study sample by selected variables.

Variables	Adjusted OR (95% CI)	*p* ^1^
**Age group (years)**		0.187
18–20	Reference	
21–25	1.26 (0.89–1.77)	
**Sex**		0.050
Male	Reference	
Female	1.48 (1.00–2.19)	
**Faculty**		0.164
Medical	Reference	
Dental	0.78 (0.55–1.11)	
**Place of childhood residence**		0.023
Urban	Reference	
Rural	1.56 (1.06–2.28)	
**Self-assessed dental aesthetic**		0.008
Good	Reference	
Poor	1.75 (1.16–2.64)	
**Satisfaction with mouth and teeth**		
Yes	Reference	
No	2.51 (1.68–3.77)	<0.001
Difficult to answer	1.74 (1.04–2.90)	0.034
**Self-assessed oral health**		0.184
Good	Reference	
Poor	1.34 (0.87–2.05)	
**DMFT**	1.05 (1.01–1.09)	0.019
**OHI-S**	1.41 (1.00–2.00)	0.052

Abbreviations: OR odds ratio; CI confidence interval; DMFT decayed missing and filled permanent teeth; OHI-S Simplified Oral Hygiene Index. ^1^ *p* from the final multivariable binary logistic regression with backward stepwise selection of variables; Cragg & Uhler’s R square = 20.6%; Gingival Index and skipping tooth-brushing were removed from the final model.

## Supplementary Materials

The following is available online at http://www.mdpi.com/1660-4601/15/4/719/s1, Table S1: The Russian version of OHIP-14.
