# Supplementary material for: Oral Health-Related Quality of Life in Young Adults: A Survey of Russian Undergraduate Students
Source: Int J Environ Res Public Health. 2018 Apr 11;15(4):719. doi: 10.3390/ijerph15040719 (PMC5923761; doi:10.3390/ijerph15040719)
Supplement: Supplementary file 1 [file ijerph-15-00719-s001.pdf]

International Journal of
Environmental Research and Public Health

MDPI

Supplementary Materials

Oral Health-Related Quality of Life in Young Adults: A Survey of Russian Undergraduate Students

Sergei N. Drachev, Tormod Brenn and Tordis A. Trovik

Table S1. The Russian version of Oral Health Impact Profile-14 (OHIP-14)*

	никогда	почти никогда	иногда	достаточно часто	очень часто	не знаю
1. Испытываете ли Вы затруднения при произношении слов из-за проблем с зубами, слизистой оболочкой полости рта или протезами?						
2. Вы потеряли вкус к пище из-за проблем с зубами, слизистой оболочкой полости рта или протезами?						
3. Испытываете ли Вы болевые ощущения в полости рта?						
4. Вызывает ли у Вас затруднение прием пищи из-за проблем с зубами, слизистой оболочкой полости рта или протезами?						
5. Чувствуете ли Вы себя стесненным в общении с людьми из-за проблем с зубами, слизистой оболочкой полости рта или протезами?						
6. Испытываете ли Вы неудобства из-за проблем с зубами, слизистой оболочкой полости рта или протезами?						
7. Питаетесь ли Вы неудовлетворительно из-за проблем с зубами, слизистой оболочкой полости рта или протезами?						
8. Приходится ли Вам прерывать прием пищи из-за проблем с зубами, слизистой оболочкой полости рта или протезами?						
9. Мешают ли Вам проблемы с зубами, слизистой оболочкой полости рта или протезами отдыхать, расслабляться?						

10. Ставят ли Вас проблемы с зубами, слизистой оболочкой полости рта или протезами в неловкое положение?						
11. Приводят ли Вас проблемы с зубами, слизистой оболочкой полости рта или протезами к повышенной раздражительности при общении с людьми?						
12. Испытываете ли Вы затруднения в обычной работе из-за проблем с зубами, слизистой оболочкой полости рта или протезами?						
13. Становится ли Ваша жизнь менее интересной из-за проблем с зубами, слизистой оболочкой полости рта или протезами?						
14. Приходится ли Вам полностью «выпадать из жизни» из-за проблем с зубами, слизистой оболочкой полости рта или протезами?						

* the items were taken from the paper Barer, G.M.; Gurevich, K.G.; Smirniagina, V.V.; Fabrikant, E.G. Validation of oral health impact profile (OHIP) quality of life questionnaire in Russian patients with evidence of chronic generalized periodontitis. *Stomatologiia (Mosk)* **2007**, *86*, 27-30. (In Russian).
